# Supplementary material for: Clinical carbapenem-resistant Enterobacterales in a University Hospital in Dakar, Senegal: genomic insights into Enterobacter hormaechei ST182 strains carrying blaNDM-5 and blaOXA-48 genes
Source: Microbiol Spectr. 2025 Sep 16;13(10):e00780-25. doi: 10.1128/spectrum.00780-25 (PMC12502527; doi:10.1128/spectrum.00780-25)
Supplement: Supplemental Material — Tables S1 to S3; Fig. S1 and S2. [file spectrum.00780-25-s0001.docx]

**Supplementary Material**

**Clinical carbapenem-resistant *Enterobacterales* in a university hospital in Dakar, Senegal: genomic insights into *Enterobacter* *hormaechei* ST182 strains carrying *bla*_NDM-5_ and *bla*_OXA-48_ genes**

Komla Mawunyo Dossouvi^1,2,^*, Bissoume Sambe Ba^3^, Gora Lo^1,4^, Fábio Parra Sellera^5,6^, João Pedro Rueda Furlan^7^, Antoine Culot^8^, Guillaume Abriat^8^, Adja Bousso Gueye^9^, Awa Ba-Diallo^1,4^, Assane Dieng^1^, Fatime Poulo Ly^9^, Abdoulaye Cissé^1^, Serigne Mbaye Lo Ndiaye^1^, Alioune Tine^1^, Farba Karam^1^, Habsa Diagne-Samb^1^, Safietou Ngom-Cisse^1^, Halimatou Diop-Ndiaye^1,4^, Coumba Toure-Kane^4^, Aïssatou Gaye-Diallo^1,4^, Sika Dossim^10^, Souleymane Mboup^1,4^, Cheikh Saad Bouh Boye^1^, Abdoulaye Seck^11^, Makhtar Camara^1,4^

**Affiliations:**

^1^Bacteriology-Virology laboratory, National University Hospital Aristide Le Dantec, Dakar, Senegal.

^2^Department of Microbiology, Global Health Research Institute, Lomé, Togo.

^3^WHO Country Office Senegal, World Health Organization, Dakar, Senegal.

^4^Institut de Recherche en Santé, de Surveillance Epidémiologique et de Formation (IRESSEF), Dakar, Senegal.

^5^Department of Internal Medicine, School of Veterinary Medicine and Animal Science, University of São Paulo, São Paulo, Brazil.

^6^School of Veterinary Medicine, Metropolitan University of Santos, Santos, Brazil.

^7^Paulista School of Medicine, Federal University of São Paulo, São Paulo, Brazil.

^8^Rime Bioinformatics, Palaiseau, Île-de-France, France.

^9^Pole of Microbiology, Institut Pasteur de Dakar, Dakar, Senegal.

^10^Faculté des Sciences de la Santé, Université de Kara, Kara, Togo.

^11^Medical Analysis laboratory, Institut Pasteur de Dakar, Dakar, Senegal.

***Corresponding author:** Komla Mawunyo Dossouvi, Bacteriology-Virology Laboratory, National University Hospital Aristide Le Dantec, Dakar, Senegal. E-mail: [dossouvikomlamawunyo@gmail.com](mailto:dossouvikomlamawunyo@gmail.com).

**Supplementary Table S1.** Antimicrobial susceptibility and antimicrobial resistance genes in CRE isolates

| **ID** | **Species** | **Sample** | **Department** | **Outpatient/ inpatient** | **Imipenem** | **Ertapenem** | **Meropenem** | **Ampicillin** | **Ampicillin-sulbactam** | **Amoxicillin-clavulanic acid** | **Ticarcillin-clavulanic acid** | **Ticarcillin** | **Piperacillin** |
| --- | --- | --- | --- | --- | --- | --- | --- | --- | --- | --- | --- | --- | --- |
| 45 | *K. pneumoniae* | blood | A-R | inpatient | ≥ 16; R | ≥ 8; R | ≥ 16; R | ≥ 32; R | ≥ 32; R | ≥ 32; R | ≥ 128; R | ≥ 128; R | ≥ 128; R |
| 202 | *E. hormaechei* | pus | A-R | Inpatient | ≥ 16; R | ≥ 8; R | ≥ 16; R | ≥ 32; R | ≥ 32; R | ≥ 32; R | ≥ 128; R | ≥ 128; R | ≥ 128; R |
| 254 | *K. pneumoniae* | bronchial fluid | A-R | Inpatient | ≥ 16; R | ≥ 4; R | 1; S | ≥ 32; R | ≥ 32; R | ≥ 32; R | ≥ 128; R | ≥ 128; R | ≥ 128; R |
| 265 | *E. coli* | urine | urology | outpatient | 4; I | ≥ 8; R | 1; S | ≥ 32; R | ≥ 32; R | ≥ 32; R | ≥ 128; R | ≥ 128; R | ≥ 128; R |
| 400 | *Ecc* | urine | pediatrics | Outpatient | ≥ 16; R | ≥ 8; R | ≥ 16; R | ≥ 32; R | ≥ 32; R | ≥ 32; R | ≥ 128; R | ≥ 128; R | ≥ 128; R |
| 558 | *K. pneumoniae* | bronchial fluid | A-R | Inpatient | ≥ 16; R | ≥ 8; R | ≥ 16; R | ≥ 32; R | ≥ 32; R | ≥ 32; R | ≥ 128; R | ≥ 128; R | ≥ 128; R |
| 718 | *K. pneumoniae* | urine | cardiology | Inpatient | 8; I | ≥ 4; R | 4; I | ≥ 32; R | ≥ 32; R | ≥ 32; R | ≥ 128; R | ≥ 128; R | ≥ 128; R |
| 1541 | *P. mirabilis* | blood | urology | Outpatient | 8; I | ≤ 0.5; I* | ≥ 16; R | ≥ 32; R | ≥ 32; R | ≥ 32; R | 16; R* | ≥ 128; R | ≥ 128; R |
| 3412 | *K. pneumoniae* | pus | orthopedics | Inpatient | ≥ 16; R | ≥ 8; R | ≥ 16; R | ≥ 32; R | ≥ 32; R | ≥ 32; R | ≥ 128; R | ≥ 128; R | ≥ 128; R |
| 3739 | *K. pneumoniae* | urine | urology | Outpatient | 2; S | ≥ 4; R | 4; I | ≥ 32; R | ≥ 32; R | ≥ 32; R | ≥ 128; R | ≥ 128; R | ≥ 128; R |
| 4049 | *Ecc* | pus | internal medicine | Inpatient | 8; I | ≥ 8; R | 8; I | ≥ 32; R | ≥ 32; R | ≥ 32; R | ≥ 128; R | ≥ 128; R | ≥ 128; R |
| 4416 | *K. pneumoniae* | urine | A-R | Inpatient | ≥ 16; R | ≥ 8; R | ≥ 16; R | ≥ 32; R | ≥ 32; R | ≥ 32; R | ≥ 128; R | ≥ 128; R | ≥ 128; R |
| 4853 | *E. hormaechei* | urine | orthopedics | Inpatient | ≤ 0.25; S | ≥ 2; R | 1; S | ≥ 32; R | ≥ 32; R | ≥ 32; R | ≥ 128; R | ≥ 128; R | ≥ 128; R |
| 7021 | *E. coli* | urine | pediatrics | Outpatient | ≥ 16; R | ≥ 8; R | ≥ 16; R | ≥ 32; R | ≥ 32; R | ≥ 32; R | ≥ 128; R | ≥ 128; R | ≥ 128; R |
| 7395 | *K. pneumoniae* | sputum | external | Outpatient | ≥ 16; R | ≥ 8; R | 4; I | ≥ 32; R | ≥ 32; R | ≥ 32; R | ≥ 128; R | ≥ 128; R | ≥ 128; R |
| 8276 | *Ecc* | urine | urology | Outpatient | ≥ 16; R | ≥ 8; R | ≥ 16; R | ≥ 32; R | ≥ 32; R | ≥ 32; R | ≥ 128; R | ≥ 128; R | ≥ 128; R |
| 8299 | *E. hormaechei* | urine | urology | Outpatient | ≥ 16; R | ≥ 8; R | ≥ 16; R | ≥ 32; R | ≥ 32; R | ≥ 32; R | ≥ 128; R | ≥ 128; R | ≥ 128; R |
| 8322 | *E. hormaechei* | pus | orthopedics | Inpatient | 8; I | ≥ 8; R | 8; I | ≥ 32; R | ≥ 32; R | 16; R | 64; R | ≥ 128; R | ≥ 128; R |
| 8381 | *K. pneumoniae* | urine | urology | Outpatient | 8; I | 4; R | 4; I | ≥ 32; R | ≥ 32; R | 16; R | 64; R | ≥ 128; R | ≥ 128; R |
| 8438 | *K. pneumoniae* | pus | urology | Outpatient | 8; I | ≥ 8; R | 8; I | ≥ 32; R | ≥ 32; R | 16; R | 32; R | ≥ 128; R | ≥ 128; R |
| 8848 | *K. pneumoniae* | urine | urology | Outpatient | ≥ 16; R | 4; R | ≥ 16; R | ≥ 32; R | ≥ 32; R | 16; R | 32; R | ≥ 128; R | ≥ 128; R |
| 9112 | *K. pneumoniae* | urine | urology | Outpatient | 2; S | ≥ 4; R | 4; I | ≥ 32; R | ≥ 32; R | ≥ 32; R | ≥ 128; R | ≥ 128; R | ≥ 128; R |
| 9304 | *K. pneumoniae* | pus | external | Outpatient | ≥ 16; R | 4; R | 8; I | ≥ 32; R | ≥ 32; R | ≥ 32; R | ≥ 128; R | ≥ 128; R | ≥ 128; R |
| 9829 | *K. pneumoniae* | urine | urology | Outpatient | ≥ 16; R | 4; R | 4; I | ≥ 32; R | ≥ 32; R | ≥ 32; R | ≥ 128; R | ≥ 128; R | ≥ 128; R |
| 11250 | *K. pneumoniae* | urine | urology | Outpatient | ≥ 16; R | ≥ 8; R | ≥ 16; R | ≥ 32; R | ≥ 32; R | ≥ 32; R | ≥ 128; R | ≥ 128; R | ≥ 128; R |
| 12034 | *K. pneumoniae* | urine | nephrology | Inpatient | 8; I | 4; R | 8; I | ≥ 32; R | ≥ 32; R | ≥ 32; R | ≥ 128; R | ≥ 128; R | ≥ 128; R |
| 12617 | *K. pneumoniae* | pus | internal medicine | Inpatient | ≥ 16; R | 4; R | 8; I | ≥ 32; R | ≥ 32; R | ≥ 32; R | ≥ 128; R | ≥ 128; R | ≥ 128; R |
| 12882 | *E. coli* | urine | urology | Outpatient | ≥ 16; R | ≥ 8; R | ≥ 16; R | ≥ 32; R | ≥ 32; R | ≥ 32; R | ≥ 128; R | ≥ 128; R | ≥ 128; R |
| Each antibiotic box includes the MIC (µg/ml) and its interpretation (MIC; Interpretation); R, resistant; S, susceptible; I, intermediate; PPT, piperacillin-tazobactam; SXT, trimethoprim-sulfamethoxazole; *Ecc*, *Enterobacter cloacae* complex; *K. pneumoniae*, *Klebsiella pneumoniae*; *E. coli*, *Escherichia coli*; *P. mirabilis*, *Proteus mirabilis*; A-R, anesthesiology-reanimation; *, interpretation by the Advanced Expert System of Vitek-2; Highlighted in green, isolates subjected to whole-genome sequencing; Highlighted in gray, presence of AMR gene | | | | | | | | | | | | | |

**Supplementary Table S1.** Antimicrobial susceptibility and antimicrobial resistance genes in CRE isolates (Continued)

| **ID** | **Species** | **PPT** | **Cefalotin** | **Cefoxitin** | **Cefotaxime** | **Cefuroxime** | **Cefuroxime axetil** | **Cefixime** | **Ceftriaxone** | **Ceftazidime** | **Cefepime** | **Aztreonam** | **Amikacin** | **Gentamicin** | **Tobramycin** |
| --- | --- | --- | --- | --- | --- | --- | --- | --- | --- | --- | --- | --- | --- | --- | --- |
| 45 | *K. pneumoniae* | ≥ 128; R | ≥ 64; R | ≥ 64; R | ≥ 64; R | ≥ 64; R | ≥ 64; R | ≥ 4; R | ≥ 64; R | ≥ 64; R | ≥ 64; R | ≥ 64; R | 16; I | ≤ 1; S | ≥ 16; R |
| 202 | *E. hormaechei* | ≥ 128; R | ≥ 64; R | ≥ 64; R | ≥ 64; R | ≥ 64; R | ≥ 64; R | ≥ 4; R | ≥ 64; R | ≥ 64; R | ≥ 64; R | ≥ 64; R | 4; S | ≥ 16; R | ≥ 16; R |
| 254 | *K. pneumoniae* | ≥ 128; R | ≥ 64; R | ≤ 4; S | ≥ 64; R | ≥ 64; R | ≥ 64; R | ≥ 4; R | ≥ 64; R | 16; R | 2; R | ≥ 64; R | ≤ 2; S | ≤ 1; S | ≥ 16; R |
| 265 | *E. coli* | ≥ 128; R | ≥ 64; R | 8; R* | ≤ 1; R* | 8; R* | 8; R* | 0.5; R | ≤ 1; R* | ≤ 1; R* | ≤ 1; R* | ≤ 1; S | ≤ 2; S | ≤ 1; S | ≤ 1; S |
| 400 | *Ecc* | ≥ 128; R | ≥ 64; R | ≥ 64; R | ≥ 64; R | ≥ 64; R | ≥ 64; R | ≥ 4; R | ≥ 64; R | ≥ 64; R | 32; R | 16; R | 4; S | ≥ 16; R | ≥ 16; R |
| 558 | *K. pneumoniae* | ≥ 128; R | ≥ 64; R | ≥ 64; R | ≥ 64; R | ≥ 64; R | ≥ 64; R | ≥ 4; R | ≥ 64; R | ≥ 64; R | ≥ 64; R | ≥ 64; R | ≥ 64; R | ≥ 16; R | ≥ 16; R |
| 718 | *K. pneumoniae* | ≥ 128; R | ≥ 64; R | ≤ 4; R* | 32; R | ≥ 64; R | ≥ 64; R | ≥ 4; R | ≥ 64; R | 8; R | 2; R* | 16; R | ≤ 2; S | ≥ 16; R | ≥ 16; R |
| 1541 | *P. mirabilis* | ≥ 128; R | ≥ 64; R | ≥ 64; R | ≥ 64; R | ≥ 64; R | ≥ 64; R | ≥ 4; R | 32; R | 16; R | 4; R* | ≤ 1; S | ≤ 2; S | ≥ 16; R | 4; R* |
| 3412 | *K. pneumoniae* | ≥ 128; R | ≥ 64; R | ≥ 64; R | ≥ 64; R | ≥ 64; R | ≥ 64; R | ≥ 4; R | ≥ 64; R | ≥ 64; R | ≥ 64; R | ≥ 64; R | 16; I | ≥ 16; R | ≥ 16; R |
| 3739 | *K. pneumoniae* | ≥ 128; R | ≥ 64; R | ≥ 64; R | ≥ 64; R | ≥ 64; R | ≥ 64; R | ≥ 4; R | ≥ 64; R | ≥ 64; R | ≥ 64; R | ≥ 64; R | 8; I* | ≥ 16; R | ≥ 16; R |
| 4049 | *Ecc* | ≥ 128; R | ≥ 64; R | 16; R | ≥ 64; R | ≥ 64; R | ≥ 64; R | ≥ 4; R | ≥ 64; R | ≥ 64; R | 32; R | ≥ 64; R | 4; S | ≥ 16; R | ≥ 16; R |
| 4416 | *K. pneumoniae* | ≥ 128; R | ≥ 64; R | ≥ 64; R | ≥ 64; R | ≥ 64; R | ≥ 64; R | ≥ 4; R | ≥ 64; R | ≥ 64; R | ≥ 64; R | ≥ 64; R | 4; S | ≥ 16; R | ≥ 16; R |
| 4853 | *E. hormaechei* | ≥ 128; R | ≥ 64; R | ≥ 64; R | ≥ 64; R | ≥ 64; R | ≥ 64; R | ≥ 4; R | ≥ 64; R | ≥ 64; R | ≥ 64; R | ≥ 64; R | ≤ 2; S | ≥ 16; R | 8; R |
| 7021 | *E. coli* | ≥ 128; R | ≥ 64; R | ≥ 64; R | ≥ 64; R | ≥ 64; R | ≥ 64; R | ≥ 4; R | ≥ 64; R | ≥ 64; R | ≥ 64; R | ≥ 64; R | 8; I* | ≥ 16; R | ≥ 16; R |
| 7395 | *K. pneumoniae* | ≥ 128; R | ≥ 64; R | ≥ 64; R | ≥ 64; R | ≥ 64; R | ≥ 64; R | ≥ 4; R | ≥ 64; R | 16; R | 2; R* | 16; R | ≤ 2; S | ≤ 1; S | ≤ 1; S |
| 8276 | *Ecc* | ≥ 128; R | ≥ 64; R | ≥ 64; R | ≥ 64; R | ≥ 64; R | ≥ 64; R | ≥ 4; R | ≥ 64; R | ≥ 64; R | ≥ 64; R | ≥ 64; R | 16; I | ≥ 16; R | ≥ 16; R |
| 8299 | *E. hormaechei* | ≥ 128; R | ≥ 64; R | ≥ 64; R | ≥ 64; R | ≥ 64; R | ≥ 64; R | ≥ 4; R | ≥ 64; R | ≥ 64; R | ≥ 64; R | ≥ 64; R | ≤ 2; S | ≥ 16; R | 8; R |
| 8322 | *E. hormaechei* | ≥ 128; R | ≥ 64; R | 32; R | ≥ 64; R | ≥ 64; R | ≥ 64; R | 2; R | ≥ 64; R | 32; R | 32; R | 32; R | 8; S | 8; R | ≤ 1; S |
| 8381 | *K. pneumoniae* | ≥ 128; R | ≥ 64; R | ≤4; R* | 32; R | ≥ 64; R | ≥ 64; R | ≥ 4; R | ≥ 64; R | 16; R | 2; R* | 16; R | ≤ 2; S | ≥ 16; R | ≥ 16; R |
| 8438 | *K. pneumoniae* | ≥ 128; R | ≥ 64; R | 8; R* | 32; R | ≥ 64; R | ≥ 64; R | ≥ 4; R | ≥ 64; R | 16; R | 4; R* | 16; R | ≤ 2; S | ≥ 16; R | 8; R |
| 8848 | *K. pneumoniae* | ≥ 128; R | ≥ 64; R | 16; R* | 32; R | ≥ 64; R | ≥ 64; R | ≥ 4; R | ≥ 64; R | 16; R | 4; R* | 16; R | 4; S | ≥ 16; R | ≥ 16; R |
| 9112 | *K. pneumoniae* | ≥ 128; R | ≥ 64; R | ≥ 64; R | ≥ 64; R | ≥ 64; R | ≥ 64; R | ≥ 4; R | ≥ 64; R | ≥ 64; R | 32; R | ≥ 64; R | 4; S | ≥ 16; R | ≥ 16; R |
| 9304 | *K. pneumoniae* | ≥ 128; R | ≥ 64; R | 4; S | ≥ 64; R | ≥ 64; R | ≥ 64; R | ≥ 4; R | ≥ 64; R | 16; R | 4; I | 32; R | 4; S | ≤ 1; S | ≥ 16; R |
| 9829 | *K. pneumoniae* | ≥ 128; R | ≥ 64; R | 4; S | 2; R* | ≥ 64; R | ≥ 64; R | ≥ 4; R | ≤1; R* | 4; R* | ≤1; R* | ≤ 1; S | ≤ 2; S | ≤ 1; S | ≤ 1; S |
| 11250 | *K. pneumoniae* | ≥ 128; R | ≥ 64; R | ≥ 64; R | ≥ 64; R | ≥ 64; R | ≥ 64; R | ≥ 4; R | ≥ 64; R | ≥ 64; R | ≥ 64; R | ≥ 64; R | 16; I | ≥ 16; R | ≥ 16; R |
| 12034 | *K. pneumoniae* | ≥ 128; R | ≥ 64; R | ≤4; R* | ≥ 64; R | ≥ 64; R | ≥ 64; R | ≥ 4; R | ≥ 64; R | 16; R | 2; R* | ≥ 64; R | ≤ 2; S | ≥ 16; R | ≥ 16; R |
| 12617 | *K. pneumoniae* | ≥ 32; R | ≥ 64; R | ≤4; R* | ≥ 64; R | ≥ 64; R | ≥ 64; R | ≥ 4; R | ≥ 64; R | 16; R | 2; R* | 16; R | ≤ 2; S | ≤ 1; S | ≤ 1; S |
| 12882 | *E. coli* | ≥ 128; R | ≥ 64; R | ≥ 64; R | ≥ 64; R | ≥ 64; R | ≥ 64; R | ≥ 4; R | ≥ 64; R | ≥ 64; R | ≥ 64; R | 16; R | ≥ 16; R | ≥ 16; R | ≥ 16; R |

**Supplementary Table S1.** Antimicrobial susceptibility and antimicrobial resistance genes in CRE isolates (Continued)

| **ID** | **Species** | **Nalidixic acid** | **Ciprofloxacin** | **Ofloxacin** | **Levofloxacin** | **Moxifloxacin** | **Tetracycline** | **Tigecycline** | **Chloramphenicol** | **Colistin** | **Trimethoprim** | **SXT** | **CMY-1** | **OXA-48** | **NDM** |
| --- | --- | --- | --- | --- | --- | --- | --- | --- | --- | --- | --- | --- | --- | --- | --- |
| 45 | *K. pneumoniae* | ≥ 32; R | ≥ 4; R | ≥ 8; R | ≥ 8; R | ≥ 8; R | 4; R | 2; I | 16; R | ≤ 0.5; S | ≤ 0.5; S | ≤ 20; S |  |  |  |
| 202 | *E. hormaechei* | ≥ 32; R | ≥ 4; R | ≥ 8; R | ≥ 8; R | ≥ 8; R | ≥ 16; R | 2; I | ≥ 64; R | ≤ 0.5; S | ≥ 16; R | ≥ 320; R |  |  |  |
| 254 | *K. pneumoniae* | ≥ 32; R | ≥ 4; R | 2; R | 1; S | 2; R | ≥ 16; R | 2; I | 4; S | ≤ 0.5; S | ≥ 16; R | ≥ 320; R |  |  |  |
| 265 | *E. coli* | 8; R | 0.5; S | 1; I | 1; S | 1; I | ≥ 16; R | ≤ 0.5; S | ≤ 2; S | ≤ 0.5; S | 8; R | ≥ 320; R |  |  |  |
| 400 | *Ecc* | ≥ 32; R | 1; I | 2; R | 1; S | 2; R | ≥ 16; R | 2; I | 8; S | ≤ 0.5; S | ≥ 16; R | 40; S |  |  |  |
| 558 | *K. pneumoniae* | ≥ 32; R | ≥ 4; R | ≥ 8; R | ≥ 8; R | ≥ 8; R | 2; S | ≤ 0.5; S | ≥ 64; R | ≤ 0.5; S | ≥ 16; R | ≥ 320; R |  |  |  |
| 718 | *K. pneumoniae* | 4; R* | 1; I | 2; R | 1; S | 2; R | ≥ 16; R | 1; S | ≤ 2; S | ≤ 0.5; S | ≥ 16; R | ≥ 320; R |  |  |  |
| 1541 | *P. mirabilis* | ≥ 32; R | ≥ 4; R | ≥ 8; R | ≥ 8; R | ≥ 8; R | ≥ 16; R | 4; R | ≥ 64; R | ≥ 16; R | ≥ 16; R | ≥ 320; R |  |  |  |
| 3412 | *K. pneumoniae* | ≥ 32; R | ≥ 4; R | ≥ 8; R | ≥ 8; R | ≥ 8; R | ≥ 16; R | ≤ 0.5; S | ≥ 64; R | ≤ 0.5; S | ≥ 16; R | ≥ 320; R |  |  |  |
| 3739 | *K. pneumoniae* | ≥ 32; R | ≥ 4; R | ≥ 8; R | ≥ 8; R | ≥ 8; R | ≥ 16; R | ≤ 0.5; S | 16; R | ≤ 0.5; S | ≥ 16; R | ≥ 320; R |  |  |  |
| 4049 | *Ecc* | ≥ 32; R | ≥ 4; R | ≥ 8; R | ≥ 8; R | ≥ 8; R | ≤ 1; S | ≤ 0.5; S | ≤ 2; S | ≤ 0.5; S | ≥ 16; R | ≥ 320; R |  |  |  |
| 4416 | *K. pneumoniae* | ≥ 32; R | ≥ 4; R | ≥ 8; R | ≥ 8; R | ≥ 8; R | ≥ 16; R | 8; R | 16; R | ≤ 0.5; S | ≥ 16; R | ≥ 320; R |  |  |  |
| 4853 | *E. hormaechei* | 8; S | 0.5; S | 2; R | 1; S | 2; R | 2; S | 1; S | 8; S | ≤ 0.5; S | ≥ 16; R | ≥ 320; R |  |  |  |
| 7021 | *E. coli* | ≥ 32; R | ≥ 4; R | ≥ 8; R | ≥ 8; R | ≥ 8; R | ≥ 16; R | ≤ 0.5; S | 8; S | ≤ 0.5; S | ≥ 16; R | ≥ 320; R |  |  |  |
| 7395 | *K. pneumoniae* | 16; R* | 0.5; S | 2; R | 1; S | 2; R | ≤ 1; S | ≤ 0.5; S | 4; S | ≤ 0.5; S | ≥ 16; R | ≥ 320; R |  |  |  |
| 8276 | *Ecc* | ≥ 32; R | ≥ 4; R | ≥ 8; R | ≥ 8; R | ≥ 8; R | ≥ 16; R | 2; I | ≥ 64; R | ≤ 0.5; S | ≥ 16; R | ≥ 320; R |  |  |  |
| 8299 | *E. hormaechei* | ≥ 32; R | ≥ 4; R | ≥ 8; R | ≥ 8; R | ≥ 8; R | ≥ 16; R | 8; R | 16; R | ≤ 0.5; S | ≥ 16; R | ≥ 320; R |  |  |  |
| 8322 | *E. hormaechei* | ≥ 32; R | 2; R | 1; R* | 4; R | 4; R | 8; R* | 1; S | 32; R | ≤ 0.5; S | ≥ 16; R | ≥ 320; R |  |  |  |
| 8381 | *K. pneumoniae* | 4; R* | 2; R | 2; R | 1; S | 1; I | ≤ 1; S | ≤ 0.5; S | ≤ 2; S | ≤ 0.5; S | ≥ 16; R | ≥ 320; R |  |  |  |
| 8438 | *K. pneumoniae* | ≥ 32; R | ≥ 4; R | ≥ 8; R | ≥ 8; R | ≥ 8; R | ≥ 16; R | 2; I | ≥ 64; R | ≤ 0.5; S | ≥ 16; R | ≥ 320; R |  |  |  |
| 8848 | *K. pneumoniae* | ≥ 32; R | ≥ 4; R | ≥ 8; R | ≥ 8; R | ≥ 8; R | ≥ 16; R | 2; I | ≥ 64; R | ≤ 0.5; S | ≥ 16; R | ≥ 320; R |  |  |  |
| 9112 | *K. pneumoniae* | ≥ 32; R | ≥ 4; R | ≥ 8; R | ≥ 8; R | ≥ 8; R | ≤ 1; S | 1; S | 8; S | ≤ 0.5; S | ≥ 16; R | ≥ 320; R |  |  |  |
| 9304 | *K. pneumoniae* | ≥ 32; R | ≥ 4; R | ≥ 8; R | ≥ 8; R | ≥ 8; R | ≥ 16; R | 2; I | 16; R | ≤ 0.5; S | ≥ 16; R | ≥ 320; R |  |  |  |
| 9829 | *K. pneumoniae* | 8; R* | ≤ 0.25; S | 1; I | 1; S | 2; R | ≤ 1; S | 1; S | ≤ 2; S | ≤ 0.5; S | ≥ 16; R | ≥ 320; R |  |  |  |
| 11250 | *K. pneumoniae* | ≥ 32; R | ≥ 4; R | ≥ 8; R | ≥ 8; R | ≥ 8; R | ≥ 16; R | 2; I | 16; R | ≤ 0.5; S | ≥ 16; R | ≥ 320; R |  |  |  |
| 12034 | *K. pneumoniae* | 8; R* | 1; I | 2; R | 1; S | 2; R | ≥ 16; R | 1; S | 4; S | ≤ 0.5; S | ≥ 16; R | ≥ 320; R |  |  |  |
| 12617 | *K. pneumoniae* | 4; R* | 0.5; S | 2; R | 1; S | 2; R | ≤ 1; S | ≤ 0.5; S | ≥ 64; R | ≤ 0.5; S | ≥ 16; R | ≥ 320; R |  |  |  |
| 12882 | *E. coli* | ≥ 32; R | ≥ 4; R | ≥ 8; R | ≥ 8; R | ≥ 8; R | ≥ 16; R | ≤ 0.5; S | 16; R | ≤ 0.5; S | ≥ 16; R | ≥ 320; R |  |  |  |

**Supplementary Table S2.** Genomic data of *Enterobacter hormaechei* isolates from this study

| **Characteristic** | **Eh8322_LBHALD**  **(BioSample: SAMN36341330)** | **Eh202_LBHALD**  **(BioSample: SAMN35822989)** |
| --- | --- | --- |
| **Species** | *Enterobacter hormaechei* | *Enterobacter hormaechei* |
| **MLST** | ST182 | ST182 |
| **Genome size (bp)** | 4,705,594 | 4,791,394 |
| **CDS** | 4,505 | 4,574 |
| **rRNA** | 9 | 12 |
| **tRNA** | 72 | 78 |
| **mRNA** | 1 | 1 |
| **Antimicrobial resistance gene** | 23 | 35 |
| **Virulence gene** | 125 | 140 |
| **Plasmid** | 2 | 1 |
| **Insertion sequence element** | 6 | 16 |
| **Horizontal gene transfer region** | 24 | 36 |
| **Transposon** | 1 | 0 |
| **Integron** | 0 | 1 |

**Supplementary Table S3.** Primers used to detect antimicrobial resistance genes

| **Target gene** | **Primer sequence (5'-3')** | **Product size (bp)** | **Annealing**  **temperature (°C)** | **Reference** |
| --- | --- | --- | --- | --- |
| *bla*_NDM_ | F: GGTTTGGCGATCTGGTTTTC  R: CGGAATGGCTCATCACGATC | 621 | 52 | (1) |
| *bla*_OXA-48_ | F: TTGGTGGCATCGATTATCGG  R: ATGGAACCCACATCGACATT | 743 | 60 | (2) |
| *bla*_OXA-23_ | F: TCTGGTTGTACGGTTCAGCA  R: GCAAAAGCGACAATTTTTCC | 501 | 55 | (3) |
| *bla*_VIM_ | F: GTTTGGTCGCATATCGCAAC  R: AATGCGCAGCACCAGGATAG | 382 | 55 | (4) |
| *bla*_KPC_ | F: CTGTCTTGTCTCTCATGGCC  R: CCTCGCTGTGCTTGTCATCC | 636 | 62 | (5) |
| *bla*_CMY-1_ | F: GTGGTGGATGCCAGCATCC  R: GGTCGAGCCGGTCTTGTTGAA | 915 | 58 | (6) |

**Supplementary Fig. S1.** Prevalence of antimicrobial resistance phenotypes and antimicrobial resistance genes in CRE isolates.


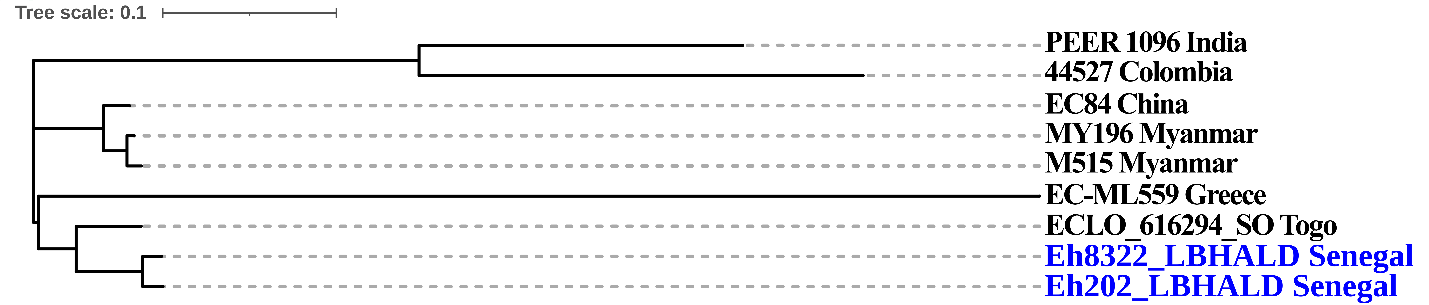


**Supplementary Fig. S2.** Phylogenetic tree of *E. hormaechei* isolates from this study and other closely related carbapenem-resistant *E. hormaechei* strains reported worldwide.

**References**

1. Nordmann P, Poirel L, Carrër A, Toleman MA, Walsh TR. 2011. How To Detect NDM-1 Producers. J Clin Microbiol 49:718–721.

2. Alousi S, Salloum T, Arabaghian H, Matar GM, Araj GF, Tokajian ST. 2018. Genomic Characterization of MDR *Escherichia coli* Harboring *bla*_OXA-48_ on the IncL/M-type Plasmid Isolated from Blood Stream Infection. BioMed Research International 2018:e3036143.

3. Naas T, N’guetta S-PA, Guessennd N, Dortet L, Makaya NPND, Tahou EJ, Abe IA, Bonnin R, Gba KMK. 2022. First Detection of Carbapenemases-Producing *Acinetobacter baumannii* and *Acinetobacter nosocomialis* in Côte d’Ivoire. J Adv Biol Biotechnol 10–23.

4. Ismail AA, Meheissen MA, Elaaty TAA, Abd-Allatif NE, Kassab HS. 2021. Microbial profile, antimicrobial resistance, and molecular characterization of diabetic foot infections in a university hospital. Germs 11:39–51.

5. Tehrani NA, Alebouyeh M, Armin S, Soleimani N, Karimi A, Shamsian B, Nazari S, Azimi L. 2023. Intestinal Carriage of Carbapenemase-Producing *Enterobacteriaceae* Members in Immunocompromised Children During COVID-19 Pandemic. Arch Pediatr Infect Dis 11:e127183.

6. Hasman H, Mevius D, Veldman K, Olesen I, Aarestrup FM. 2005. β-Lactamases among extended-spectrum β-lactamase (ESBL)-resistant *Salmonella* from poultry, poultry products and human patients in The Netherlands. J Antimicrob Chemother 56:115–121.
